# Meta-Analysis of Prognostic and Clinical Significance of CD44v6 in Esophageal Cancer

**DOI:** 10.1097/MD.0000000000001238

**Published:** 2015-08-07

**Authors:** Bangli Hu, Wei Luo, Rui-Ting Hu, You Zhou, Shan-Yu Qin, Hai-Xing Jiang

**Affiliations:** From the Department of Gastroenterology, The First Affiliated Hospital of Guangxi Medical University, Nanning, China (BH, WL, S-YQ, H-XJ); Minzu Hospital Affiliated of Guangxi Medical University, Nanning, China (R-TH); and Minerva Foundation Institute for Medical Research, Helsinki, Finland (YZ).

## Abstract

CD44v6 is a cell adhesion molecule that plays an important role in the development and progression of esophageal cancer. However, the prognostic value and clinical significance of CD44v6 in esophageal cancer remains controversial. In the present study, we aimed to clarify these relationships through a meta-analysis.

We performed a comprehensive search of studies from PubMed, EMBASE, Ovid library database, Google scholar, and Chinese National Knowledge Infrastructure databases that were published before June 2015. The odds ratio (OR) and pooled hazard ratio (HR) with the 95% confidence intervals (CI) were used to estimate the effects.

Twenty-one studies including 1504 patients with esophageal cancer were selected to assess the prognostic value and clinical significance of CD44v6 in these patients. The results showed that the expression of CD44v6 was higher in esophageal cancer tissue than in normal colorectal tissue (OR = 9.19, 95% CI = 6.30–13.42). Moreover, expression of CD44v6 was higher in patients with lymphoid nodal metastasis, compared to those without (OR = 6.91, 95% CI = 4.81–9.93). The pooled results showed that CD44v6 was associated with survival in patients with esophageal cancer (HR = 2.47, 95% CI = 1.56–3.92). No significant difference in CD44v6 expression was found in patients with different histological types and tumor stages (both *P* > 0.05). Moreover, no publication bias was found among the studies (all *P* > 0.05).

This meta-analysis demonstrates that CD44v6 is associated with the metastasis of esophageal cancer and a poor prognosis, but is not associated with the histological types and tumor stages.

## INTRODUCTION

Esophageal cancer is a common malignant tumor of the upper digestive tract. Surgical resection and other methods, such as chemotherapy and chemotherapy, have markedly improved the overall survival of patients with esophageal cancer. However, the efficacy of these methods is not satisfactory in patients with tumor metastasis. Many patients with esophageal cancer already have detectable metastasis at the time of diagnosis, including regional and/or distant metastases.^1,2^ Therefore, an investigation of the mechanism of cancer invasion and the identification of prognostic markers would be helpful for the treatment of esophageal cancer.

The loss of cell adhesion is one of currently known factors that promotes both invasion and metastasis of tumor cells. The aberrant expression or function of cell adhesion molecules results in the loss of tissue architecture, thus providing an opportunity for tumor cells to invade and progression.^3^ CD44 is a glycosylated cell surface molecule and is involved in cell–cell and cell–matrix interactions. CD44 has many variant isoforms, which are generated by alternative splicing of at least 10 exons (v1–v10). Among them, CD44v6 has been widely investigated in many tumors and reportedly has prognostic value.^4–6^ Currently, several studies have indicated a relationship between CD44v6 and the prognosis in esophageal cancer. However, the results of these studies are inconsistent.

These discrepancies among such studies may be due to the relatively small sample size. Meta-analysis is a quantitative method used to combine the results of a single study, and this approach has been successfully used for the evaluation of the prognostic indicators in patients with malignancies. Therefore, in the present study, we aimed to perform a meta-analysis of all eligible studies to clarify the relationship between CD44v6 with esophageal cancer and its prognostic value in patients with esophageal cancer.

## MATERIALS AND METHODS

### Search Strategy and Selection Criteria

This meta-analysis was conducted in accordance with the guidelines of the Preferred Reporting Items for Systematic Review and Meta-analyses statement (PRISMA). The electronic databases, including PubMed, EMBASE, Ovid library database, Google scholar, and Chinese National Knowledge Infrastructure, were searched to identify suitable literature before June 2015. The search terms included “Esophageal Neoplasms” or “Esophageal Cancer,” “CD44v6” or “CD44v6 antigen” and “Prognosis.” Only human studies were included. Conference abstracts were not selected because these studies contain insufficient data. Studies were also retrieved using the related articles function in PubMed. Moreover, the references within the identified studies were searched manually. The study was approved by the Review Boards of the First Affiliated Hospital of Guangxi Medical University.

### Inclusion and Exclusion Criteria

Studies were included in the meta-analysis if they fulfilled the following conditions: diagnosis of esophageal cancer was made using pathological examination; the association between CD44v6 expression and clinical parameters or prognosis of esophageal cancer was evaluated; and immunohistochemistry (IHC) methods were used to determine CD44v6 expression. Studies were excluded if they were review articles, case reports, or animal studies. Studies on cell lines and human xenografts were also excluded. If some studies used the same patient population, we chose the most recent study for analysis. Two reviewers assessed the eligibility of the studies independently. Disagreements between the reviewers were resolved by consensus or via consensus with a third reviewer.

### Data Extraction and Quality Assessment

The extracted data included the first author names, year of publication, country, number of male and female patients, IHC methodology (primary antibody source and dilution concentration), clinical parameters (tumor stage, lymphoid nodal metastasis, and distant metastasis), period of follow-up, and survival data. The Newcastle Ottawa Quality Assessment Scale (NOS)^7^ was used to assess and score the quality of the methodology of each study. We assessed the item of the exposed cohort, ascertainment of exposure, outcome of interest, comparability of cohorts, assessment of outcome, and adequacy of follow-up for each study. A study with a score of 6 or higher was considered as a high-quality study. Two reviewers extracted the data independently, and calculated the quality score of each study. Disagreements between the reviewers were resolved by consensus or via consensus with a third reviewer.

### Statistical Analysis

The pooled odds ratio (OR) and its 95% confidence interval (CI) were used to quantitatively determine the association between CD44v6 expression and the clinical parameters of esophageal cancer. The hazard ratio (HR) and its 95% CI were used to quantitatively evaluate the association between CD44v6 expression and patient survival. Heterogeneity among studies was assessed using Cochran's Q test and the I^2^ statistic. I^2^ values of <25% were indicative of mild heterogeneity, I^2^ values between 25% and 50% were indicative of moderate heterogeneity, and I^2^ values of >50% were indicative of significant heterogeneity. A fixed-effect model (Mantel–Haenszel method) was used to calculate parameters in cases where heterogeneity was low; in other cases, a random-effect model (DerSimonian–Laird method) was used. Sensitivity analysis was performed to test the reliability of the overall pooled results by excluding each study in turn. Publication bias was assessed using the Egger test and Begger's test. All statistical tests in this meta-analysis were performed using Stata 11.2 software (Stata Corp, College Station, TX) with 2-tailed *P* values. A *P* value of <0.05 was considered statistically significant.

## RESULTS

### Identification of Relevant Studies

**FIGURE 1:**
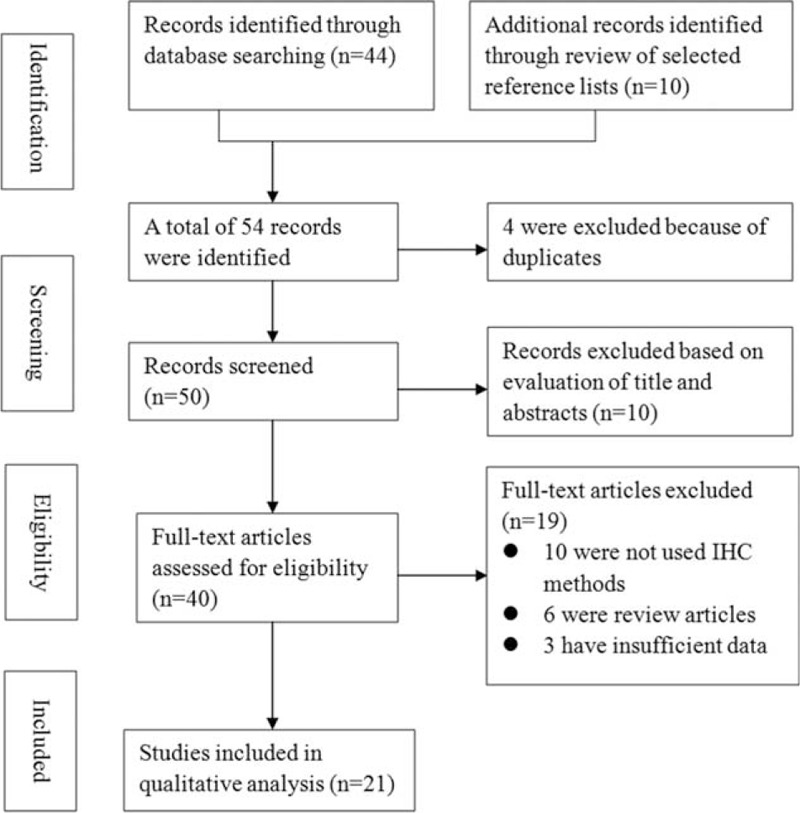
Flowchart of study selection.

The initial search yielded 38 studies that were considered eligible according to the predefined selection criteria. After screening the full text, 19 studies were excluded. Of these, 10 did not use IHC methods, 6 were review articles, and 3 had insufficient data. Finally, 21 studies^8–28^ with 1504 patients with esophageal cancer were included in the present meta-analysis. Figure 1 shows a summary of the selection process.

### Study Characteristics and Quality Assessment

All the included studies used the IHC method to determine the expression of CD44v6 in esophageal cancer tissues; 6 studies^8,12,18,20–22^ conducted a follow-up observation, but 1^8^ of these studies did not provide the follow-up data. The source of the primary antibody and the diluted concentration varied among the included studies. The quality of studies was high and the score of each study was >6. The patient demographics from each study are listed in Table 1.

**TABLE 1:**
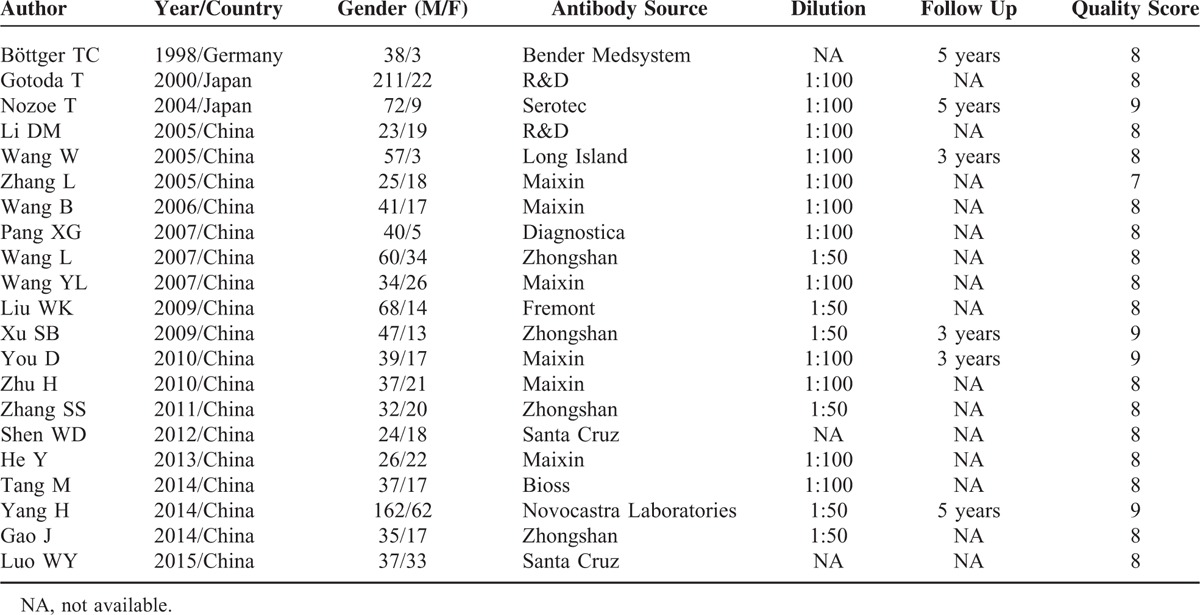
Characteristics of Selected Studies in the Meta-Analysis

### Expression of CD44v6 in Esophageal Tissue

**FIGURE 2:**
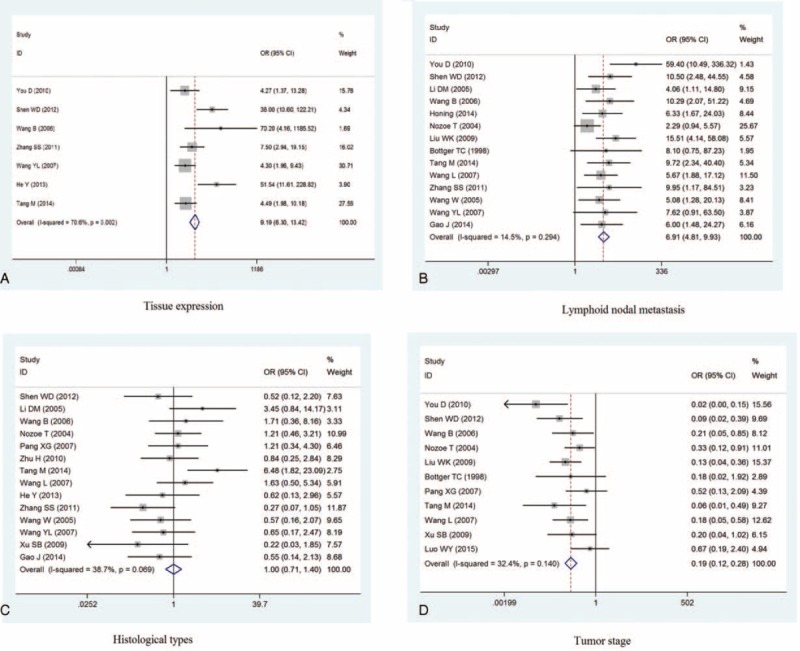
Meta-analysis of CD44v6 expression in esophageal cancer. A: esophageal cancer and normal esophageal tissue; B: lymphoid node metastasis; C: histological types; D: tumor stag; D survival time.

Seven studies,^14–16,19,22,24,26^ including 194 specimens, compared the expression of CD44v6 in esophageal cancer tissues and normal esophageal tissues. We found that the expression of CD44v6 was higher in esophageal cancer tissues than in normal esophageal tissues (OR = 9.19, 95% CI = 6.30–13.42). This result suggests that CD44v6 may be involved in the pathogenesis of esophageal cancer. However, there was significant heterogeneity across the studies (I^2^ = 70.6%, *P* = 0.002; see Figure 2A). Sensitivity analysis was thus performed by removing each study in turn; although the heterogeneity decreased, the pooled ORs remained similar to the overall results, indicating the stability of the results. The funnel plots were largely symmetrical, and no publication biases were noted across the studies (Egger test = 0.118, Begg's test = 0.063).

### Association Between CD44v6 in Esophageal Cancer and Clinical Parameters

We examined the association between CD44v6 expression and the clinical parameters of esophageal cancer. The pooled results of 14 studies^8,10–12,14–20,22,24,25^ showed that the expression of CD44v6 was higher in patients with lymphoid nodal metastasis, compared with those without (OR = 6.91, 95% CI = 4.81–9.93, I^2^ = 14.5%, *P* = 0.294). Only 2 studies^10,14^ provided data on distant metastasis, and although both these studies indicated that CD44v6 expression was higher in patients with distant metastasis compared with those without, we did not pool these data considering the lack of robustness of the results.

With regard to the histological types, the pooled results of 14 studies^10,12–20,24,26^ showed no significant difference in poorly differentiated esophageal cancer (OR = 1.00, 95% CI = 0.71–1.40, I^2^ = 38.7%, *P* = 0.069). Similar results were observed with regard to the tumor stage, which was described in 11 studies^8,11–17,20,22^ (OR = 0.19, 95% CI = 0.12–0.28; I^2^ = 32.4%, *P* = 0.140; see Figure 2B–D).

The test of publication bias showed no publication bias among the included studies with regard to the above measurements (lymphoid nodal metastasis: Egger test = 0.181, Begg's test = 0.252; tumor stage: Egger test = 0.232, Begg's test = 0.760; histological types: Egger test = 0.709, Begg's test = 0.584).

### Relationship Between CD44v6 and Esophageal Cancer Prognosis

**FIGURE 3:**
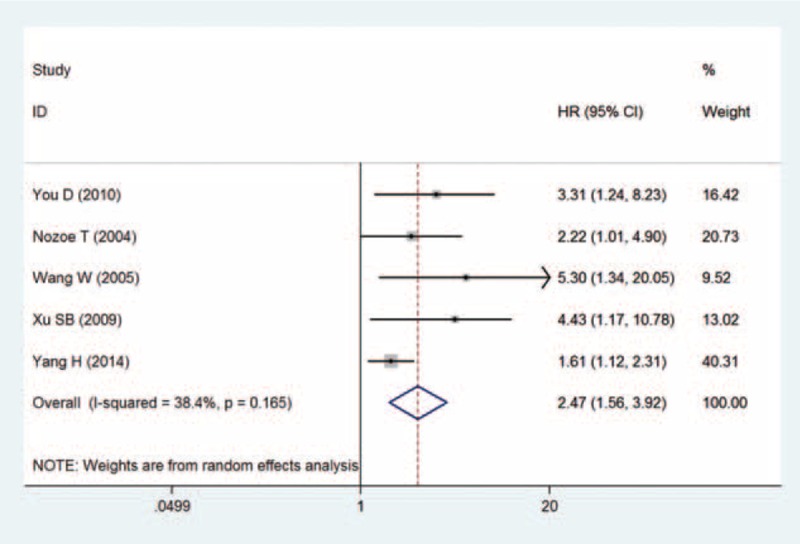
Meta-analysis of the CD44v6 expression with survival time in patients with esophageal cancer.

Five studies^12,18,20–22^ provided data on CD44v6 expression as well as survival duration of patients with esophageal cancer. The pooled results showed that high expression of CD44v6 was associated with poor prognosis in patients with esophageal cancer (HR = 2.47, 95% CI = 1.56–3.92, I^2^ = 38.4%, *P* = 0.165; see Figure 3). Moreover, the risk of publication bias was not significant across the studies (Egger test = 0.069, Begg's test = 0.089).

## DISCUSSION

Multiple factors and mechanisms are involved in the metastasis of malignant tumors, including growth factors, proteolytic enzymes penetrating the extracellular matrix, adhesion molecules, and angiogenesis factors (which vascularize the tumor in several steps throughout metastasis).^29^ Invasion and metastasis are found to be the main biological characteristics of esophageal cancer, as well as the main causes of treatment failure and mortality. CD44 is a family of cell adhesion molecules and has numerous splice variants. CD44 mediates the regulation of cell division, survival, migration, and adhesion through the binding of its major ligand, hyaluronic acid, and acts as a cellular platform for growth factors and heparan-sulfate proteoglycans.^30^ Because CD44 reacts with the extracellular matrix, studies suggest that its expression is related to metastatic potential, prognosis, and the biologic properties of human malignancies.^31,32^

CD44v6—a CD44 variant—changes the composition and function of adhesion molecules. Studies indicated that increased CD44v6 expression would induce a high metastatic potential in some tumors, such as nonsmall cell lung cancer,^33^ gastric cancer,^34^ and pharyngolaryngeal cancer.^35^ However, with regard to esophageal cancer, the reports have been inconsistent. For example, Shen et al^14^ reported that CD44v6 expression was significantly higher in esophageal cancer tissue than in adjacent normal tissue, and was correlated with differentiation, distant metastases, and TNM stage (*P* < 0.05), but not with lymph node metastasis. Moreover, Nozoe et al^12^ showed that high expression of CD44v6 was associated with lymph node metastasis. More recently, Yang et al^21^ reported that there were no significant correlations between CD44v6 expression and tumor size, tumor location, depth of invasion, and pathological stage. These inconsistent results suggest that further study is required to clarify these associations.

In the present meta-analysis, the pooled results suggested that the expression of CD44v6 was higher in esophageal cancer tissue than in normal tissue; however, there was significant heterogeneity across the studies, which will undermine the robustness of the conclusion. We postulated that the source of heterogeneity may include factors such as the use of primary antibodies from different companies, different tumor staging methods, and different evaluation standards. Our results also found that the high expression of CD44v6 was associated with poor survival in esophageal cancer patients with lymphoid nodal metastasis, which suggests that the abnormal expression of CD44v6 in tumor cells may enhance their potential for metastasis in the regional lymph nodes. However, no association was observed between the expression of CD44v6 and the tumor stage and histological types, indicating that CD44v6 may not be involved in the biological function of tumor cells but may promote the metastasis of tumor cells. However, the exact mechanisms of these effects are not clear and need to be investigated further.

To our knowledge, this is the first meta-analysis to investigate the prognostic value and clinical significance of CD44v6 expression in esophageal cancer. The large sample size of our study enhanced the statistical power, thus enabling the detection of a more stable association and providing a more reliable estimation than any previous individual study. In addition, the sensitivity analysis showed consistent results, and no evidence of publication bias was observed, suggesting the robustness of the results. However, the finding of this study should be interpreted while considering certain limitations. First, the cut-off value for defining high CD44v6 expression varied among the eligible studies. Second, the heterogeneity present in the methods of measurement of CD44v6 expression in esophageal cancer tissue may decrease the robustness of our conclusion. Third, due to the lack of sufficient data, we could not analyze the association between CD44v6 expression and distant metastasis, which could provide more information to better understand the role of CD44v6 in esophageal cancer. Hence, additional well-designed studies with larger sample sizes are needed to provide a more comprehensive evaluation of the prognostic value of CD44v6 expression in patients with esophageal cancer.

In conclusion, this meta-analysis indicates that high expression of CD44v6 is associated with a poor survival and lymphoid nodal metastasis in patients with esophageal cancer. However, in order to achieve a more comprehensive evaluation of the prognostic role of CD44v6 expression in patients with esophageal cancer, more well-designed studies with larger sample sizes are warranted.
